# A literature therapeutic group at a psychiatric closed-unit

**DOI:** 10.1192/j.eurpsy.2021.2076

**Published:** 2021-08-13

**Authors:** H. Yaniv, D. Shalom Nimni, S. Raskin

**Affiliations:** Closed Unit, Jerusalem Mental Health Center, Kfar Shaul Hospital, Israel, Jerusalem, Israel

**Keywords:** Psychotic disorders, Literature, Therapeutic Group, Psychiatric Closed-Unit

## Abstract

**Introduction:**

This lecture will present a therapeutic group that took place at a closed-unit in a psychiatric hospital. The members of this group were patients suffering from psychotic disorders

**Objectives:**

Patients suffering from psychotic disorders.

**Methods:**

The patients had difficulty in organizing their thoughts as well as with the expression of their internal-world and emotions. Moreover, they were suspected of the units’ staff members. These circumstances led us to create a theme group that combines a verbal-affective metaphoric instrument - literature. Art, such as literature, represents the mind of its creator and when incorporated into the therapeutic process, can serve as a third-voice - a symbolic language that conveys an idea indirectly and arouses the patient’s personal associations and emotions. The use of literature, while relating to content that aroused from a poem or a short story, led to a connection or an identification with the emotion expressed in the writing stimuli or in opposition to it, and from there to a projection of the internal world of the patient.

**Results:**

Through the possibility of alternating between proximity and distance, regard the metaphoric instrument, patients could organize their associations and emotions and express them in a more beneficent way – “normalization” of the cognitive and expressive process.

**Conclusions:**

The analysis of the different group’s sessions, points to the potential of using literature in a therapeutic group with patients in their acute state, at the closed-unit. Examples of verbal reports from different group settings, in which literature was used, will be presented.

**Disclosure:**

No significant relationships.

